# Exciton Emission Intensity Modulation of Monolayer MoS_2_ via Au Plasmon Coupling

**DOI:** 10.1038/srep41175

**Published:** 2017-01-30

**Authors:** B. Mukherjee, N. Kaushik, Ravi P. N. Tripathi, A. M. Joseph, P. K. Mohapatra, S. Dhar, B. P. Singh, G. V. Pavan Kumar, E. Simsek, S. Lodha

**Affiliations:** 1 Department of Electrical Engineering, Indian Institute of Technology Bombay, Mumbai, Maharashtra 400076, India; 2 Photonics and Optical Nanoscopy Laboratory, Physics Division and Center for Energy Science, h-cross, Indian Institute of Science Education and Research, Pune 411008, India; 3 Department of Physics, Indian Institute of Technology Bombay, Mumbai, Maharashtra 400076, India; 4 Department of Electrical and Computer Engineering, School of Engineering and Applied Science, The George Washington University, Washington, D.C. 20052, USA

## Abstract

Modulation of photoluminescence of atomically thin transition metal dichalcogenide two-dimensional materials is critical for their integration in optoelectronic and photonic device applications. By coupling with different plasmonic array geometries, we have shown that the photoluminescence intensity can be enhanced and quenched in comparison with pristine monolayer MoS_2_. The enhanced exciton emission intensity can be further tuned by varying the angle of polarized incident excitation. Through controlled variation of the structural parameters of the plasmonic array in our experiment, we demonstrate modulation of the photoluminescence intensity from nearly fourfold quenching to approximately threefold enhancement. Our data indicates that the plasmonic resonance couples to optical fields at both, excitation and emission bands, and increases the spontaneous emission rate in a double spacing plasmonic array structure as compared with an equal spacing array structure. Furthermore our experimental results are supported by numerical as well as full electromagnetic wave simulations. This study can facilitate the incorporation of plasmon-enhanced transition metal dichalcogenide structures in photodetector, sensor and light emitter applications.

Two-dimensional (2D) transition metal dichalcogenides (TMDCs) hold significant promise for future optoelectronic applications^1^,^2^. Luminescence from TMDCs can be obtained using optical excitation as well as electrical pumping^3^,^4^. Also, unique optical properties of localized surface plasmon resonance (LSPR) structures have led to demonstration of simultaneous enhancement in excitation and emission fields as well as enhancement in light emitting diode performance^5^,^6^. Wavelength of the surface plasmon resonance (SPR) is determined by the dielectric function of the active material and its surroundings^7^,^8^. In recent decades, several studies have been reported on LSPR for different shapes of Au nanostructures^9^ on various substrates such as quartz^10^, SiO_2_/Si^11^, graphene coated substrates^2^,^12^,^13^, and dielectric layer coated substrates^14^,^15^, etc. It has been seen that the figure of merit of an LSPR sensor is highly dependent on the shape of the metal nanopillars (NPs), arrangement of the NPs on the substrate^1^,^10^,^15^,^16^, and the dielectric pillars that lift the metal nanostructures above the substrate^10^. It has been demonstrated earlier that a sharp nanocone (NC) geometry gives rise to focused electromagnetic fields at the cone apexes resulting in efficient control and manipulation of optical radiation incident on the nanostructures^17^,^18^. The sharp tips of the NCs give rise to ultrahigh surface enhanced Raman scattering from the focused fields at the cone apexes^18^. LSPR also offers the ability to control the emission from quantum emitters due to high Purcell enhancement by allowing additional local density of states. 2D atomically thin TMDCs can act as a platform to investigate and exploit exciton-plasmon coupling that can help to realize novel 2D plasmonic-polaritonic devices. Visible range excitonic photoluminescence (PL) peaks from TMDCs can couple strongly with the SPR band resulting from metal plasmonic arrays fabricated on atomically thin TMDCs resulting in sharp excitonic emission peaks due to the TMDC and an interband absorption peak due to the SPR. This coupling can be modulated by engineering the arrangement of Au cone shaped nanopillars. Apart from modulating exciton emission intensity, the patterned plasmonic array creates localized fields which help the coupled TMDC layer to achieve higher optical absorption^19^. Arrangement of Au NCs on top of the layered TMDC film can lead to interesting effects like doping, modification of the field intensity distribution, a large PL enhancement, and surface-enhanced Raman scattering intensity at room temperature^20^,^21^,^22^,^23^,^24^. Tuning of the exciton emission of monolayer TMDCs has been studied previously by utilizing a gating electric field, a spacer layer between the TMDC and the plasmonic array with different thickness, varying plasmonic array geometry, varying shape of the metal nanostructures and varying surface coverage^25^,^26^,^27^,^28^,^29^. The tunability of luminescence intensity makes thin TMDCs promising for applications in sensing and as light emitting diodes.

Defect-mediated nonradiative recombination in TMDCs is the cause of low quantum yield. It has been observed that without a separation layer between monolayer (ML) molybdenum disulfide (MoS_2_) and a plasmonic array, the excitons created by illumination get dissociated and the electrons are transferred to the metal, which effectively reduces the radiative emission rate of the excitonic transition of the monolayer MoS_2_^30^. On the other hand due to the low quantum yield of ML MoS_2_, the quenching of PL intensity due to direct contact of metal plasmonic array can be overcome by radiative recombination^25^. Plasmonic arrays integrated on top of ML MoS_2_ have shown strong exciton-plasmon coupling involving three types of resonances-corresponding to (i) MoS_2_ excitons, (ii) LSPR of individual nanostructures, and (iii) lattice resonance of the nanostructure array^23^,^24^. Control of the SPR band to match the excitonic peak of the TMDC is important to achieve maximum efficiency. Recently, a few research groups have demonstrated the PL enhancement of ML MoS_2_ as well as some other monolayer TMDCs using SPR of a specific plasmonic array^20^,^25^,^26^,^27^,^31^,^32^. However introduction of asymmetric plasmonic NC arrays on ML MoS_2_ and tunability of the PL intensity with polarization angle of the incident radiation has not been demonstrated previously to the best of our knowledge.

In this report, we have experimentally and numerically studied the effect of gold (Au) plasmonic NC arrays arranged on top of an atomically thin MoS_2_ layer on a SiO_2_/Si substrate for two different array periodicities along x- and y- axes of the NC arrays. We have demonstrated both enhancement and quenching of photoluminescence of monolayer MoS_2_ by exploiting the coupling with NC arrays. Approximately 3.5-times enhanced PL has been observed with a double periodicity Au NC array. On the other hand we demonstrated quenching of PL when the Au NC array has equal spacing along both axes.

Tuning the luminescence intensity by changing the angle of polarization of the incident excitation light would add an additional degree of freedom and flexibility to develop SPR-exciton based nanosensors. In this work, asymmetric spacing along x- and y- axes (double periodicity along y axis) in the plasmonic array is used to tune the intensity of induced electric field (E-field) inside ML MoS_2_ under irradiation of incident laser excitation with different polarization angles. As a result, we are able to tune the intensity of exciton originated PL by varying the incident light polarization for the asymmetric Au NC array. Further, we show that PL intensity modulation is controlled via both optical field enhancement and spontaneous emission rate enhancement. Enhancement and quenching of luminescence from plasmonic array on ML MoS_2_ structure and its far field radiation profile have been studied numerically.

## Results and Discussion

**Figure 1:**
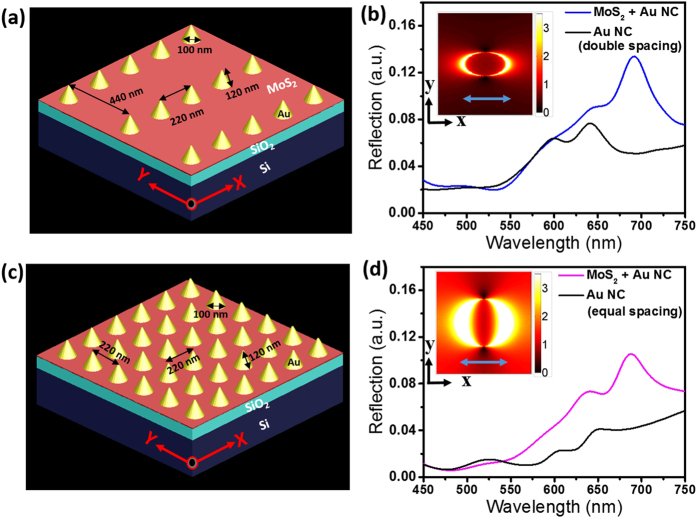
(**a,c**) Schematic representations of the double and equal spacing array structures on top of ML MoS_2_, respectively. Dimensions are not to scale. Simulated reflectance spectra when polarized plane wave is incident along x-axis: (**b,d**) for the structures in (**a** and **c**), respectively. Insets in (**b** and **d**) show the localized E-field color plot (at 680 nm) at the interface plane between Au NC and SiO_2_ layer for double and equal spacing array, respectively.

**Figure 2:**
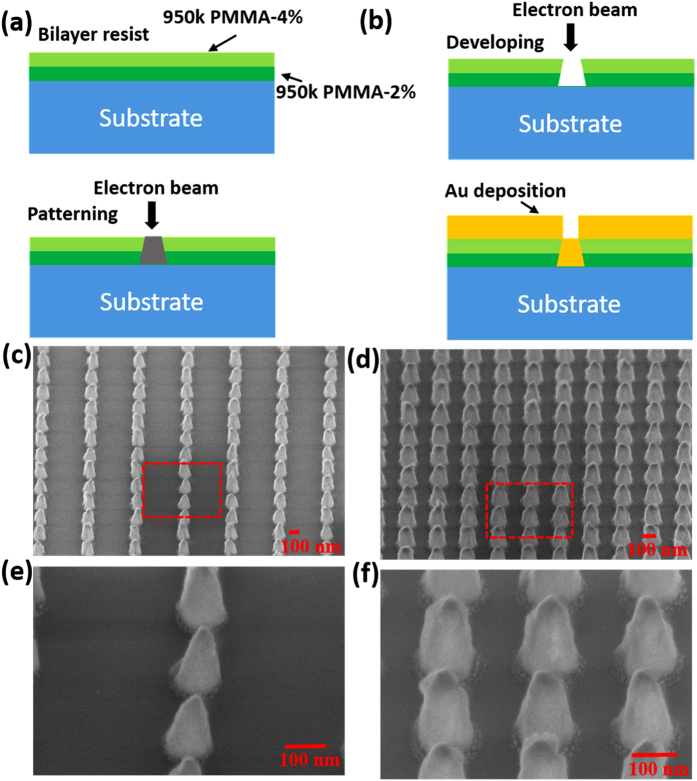
(**a,b**) Schematic process flow diagrams describing fabrication of the Au NC plasmonic array on a SiO_2_ (90 nm)/Si substrate. (**c,d**) Low magnification tilted (50°) scanning electron microscopy (SEM) images of two different Au nanocone arrays. (**e,f**) Higher magnification images from the red dotted boxes shown in (**c,d**).

In our fabricated structure, the apex tip of the NC is vertically outward and the circular base is placed on top of ML MoS_2_. Double spacing Au array- lateral periodicity along y- axis is double than the lateral periodicity along x-axis, is shown in Fig. 1a. Equal spacing array- lateral periodicity along x- and y- axes is the same (Fig. 1c). Zoomed in scanning electron microscope (SEM) images of the two different fabricated Au NC arrays are shown in Fig. 2e and f, respectively. We have obtained Au NCs with an average bottom diameter of ~113 nm ( ± 2 nm) and a height of ~120 nm ( ± 10 nm) (Figures S1, S2). In simulation design, an array of Au NCs with bottom diameter of 100 nm, top diameter of 10 nm and height of 120 nm is placed on top of SiO_2_/Si substrate where spacings along x- and y-axes are 220 nm and 440 nm, respectively (Fig. 1a). To illustrate how the double and equal spacing plasmonic arrays tune ML MoS_2_ response in the excitation and emission bands, we have simulated reflectance spectra of both the designed plasmonic array structure with and without ML MoS_2_ as shown in Fig. 1b,d. Plane polarized wave along x-axis of the structure is used as incident excitation. Double spacing plasmonic array structure without MoS_2_ has higher impact on the reflection spectra at both, excitation wavelength (~532 nm) and emission wavelength (~680 nm) as compared with equal spacing plasmonic array. It can be noted that signature of the main peaks in the absorption spectrum of ML MoS_2_ at ~605 nm and ~660 nm corresponding to ‘B’ and ‘A’ excitons, respectively, are present in the reflectance spectra of ML MoS_2_ with plasmonic array structures (Fig. 1b,d). SPR of the bare NC array has red shifted after coupling with ML MoS_2_ (index ~4.5 at 680 nm) due to increase in the optical index of the medium surrounding the NC array.

ML MoS_2_ was deposited on top of a SiO_2_/Si substrate using chemical vapour deposition (CVD) as mentioned in the experimental section. A detail description about the growth process can be found elsewhere^33^. Fabrication of Au NCs array on top of ML MoS_2_ was carried out in two steps. In the first step, alignment markers are patterned on top of the ML MoS_2_ coated substrate to identify ML MoS_2_. The second step involves patterning of the Au NC array on selected MoS_2_ flake (Fig. 2a and b). Details of the parameters used in the fabrication process are given in the experimental section. Figure 2c and d show large area SEM images of two of two different Au NC arrays corresponding to double spacing and equal spacing, respectively.

**Figure 3:**
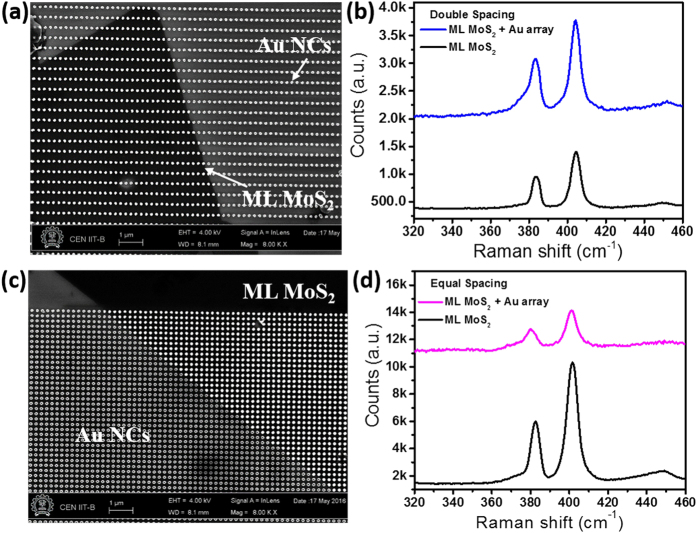
(**a**) Au plasmonic array (with periodicity of 220 nm and 440 nm along x- and y-axes, respectively) on top of monolayer (ML) MoS_2_/SiO_2_ (90 nm)/Si substrate. (**b**) Raman spectra from ML MoS_2_ with and without Au array shown in (**a**). (**c**) Au plasmonic array (with periodicity of 220 nm along both x- and y- axes) on top of ML MoS_2_/SiO_2_/Si substrate. (**d**) Raman spectra from ML MoS_2_ with and without Au array shown in (**c**).

Top view SEM images of the two Au NC arrays with different periodicities on top of ML MoS_2_ are shown in Fig. 3a and c. The fabrication process was optimized to preserve the quality of as-synthesized ML MoS_2_ during the array patterning which can be seen from the large area SEM images. The plasmonic array is placed in such a way that we can spatially differentiate between the optical response from ML MoS_2_ with and without Au array. Various regions of the MoS_2_ sample with and without plasmonic array were characterized (laser excitation wavelength = 532 nm) for quantitatively determining the layer thickness and doping effects (Fig. 3b and d) using a confocal Raman microscope (Horiba Jobin-Yvon LabRam). The Raman spectrum was collected using a 100×, 0.9 NA objective in backscattering geometry. Two prominent peaks at ~383.6 and ~404 cm^−1^ are observed (Fig. 3b), which are reported to be E^1^_2g_ and A_1g_ vibrational modes, respectively^33^, of ML MoS_2_ prepared by the CVD technique. These two main vibration modes are separated by 20.4 and 20.6 cm^−1^ for the two MoS_2_ samples confirming that these are monolayer MoS_2_. Raman spectra from the plasmonic-coupled regions have been plotted along with those from the pristine ML MoS_2_. It can be seen that the vibration band peak positions have not changed significantly after plasmonic array patterning, which confirms that there was no significant charge transfer between Au NCs and the underlying ML MoS_2_. A small shift in E^1^_2g_ mode is observed for the equal spacing Au NC array sample (Fig. 3d), which could be due to strain induced in ML MoS_2_ by the NC array^34^. However, the background scattering counts have increased^19^,^21^ for both the Au NC array structures, which could be due to plasmonic-induced enhancement of the average field intensity distribution in ML MoS_2_. Increase in intensity counts of the Raman peaks for the double spacing array coupled structure is likely due to surface enhanced Raman scattering^21^ whereas the decrease in counts and slight shift of the phonon mode (E^1^_2g_) for the equal spacing array coupled structure could be attributed to the disorder introduced in the MoS_2_ lattice by the Au NC array^34^. The peak intensity ratio (E^1^_2g_/A_1g_) has a slightly higher value for the double spacing array-coupled structure as compared with equal spacing array, which is explained later in the manuscript^26^,^34^. Raman scattering enhancement can be directly correlated with the electro-magnetic (EM) field created by localized surface plasmons of the Au NCs. Raman scattering enhancement (SE) is equal to α × |E_Au + MoS2_/E_MoS2_|^4^, where α is a proportionality constant and E is total electric field, due to an increase in both the incident laser excitation field and the emission field at the prominent Raman peak’s frequency^25^.

**Figure 4:**
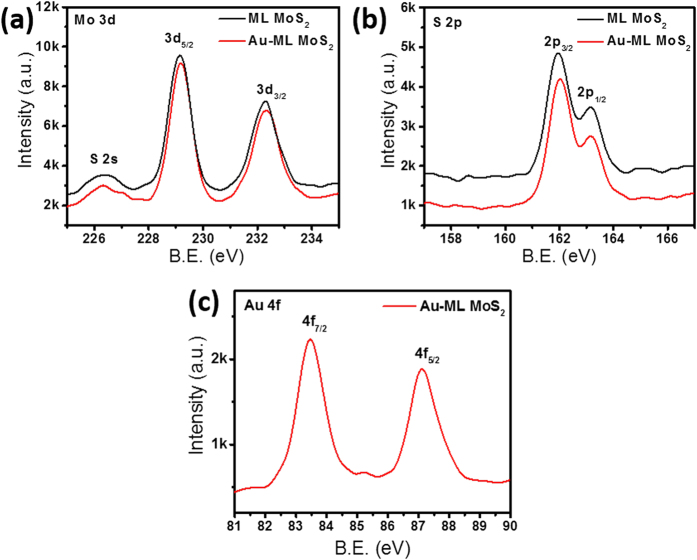
Chemical composition analysis using X-ray photoemission spectroscopy (XPS) for (**a**) molybdenum, (**b**) sulfur, and, (**c**) Au of monolayer MoS_2_ with Au plasmonic array fabricated on top.

Controlled scanning X-ray photoemission spectroscopy (XPS) was performed on ML MoS_2_ (on SiO_2_/Si substrate) with and without an Au plasmonic array (Fig. 4a–c) to gather chemical composition and doping information. Mo 3d shows two peaks at 229.2 eV and 232.3 eV corresponding to the doublet state of Mo 3d_5/2_ and Mo 3d_3/2_ (Fig. 4a). The binding energies for S 2p_3/2_ and S 2p_1/2_ are 162.0 eV and 163.3 eV, respectively (Fig. 4b). The Mo and S binding energies are in good agreement with reported values^20^, indicating that patterning of the Au plasmonic array does not change the chemical composition of monolayer MoS_2_. XPS data obtained for Au 4 f from the plasmonic array decorated ML MoS_2_ samples show that binding energies for Au 4f_7/2_ and Au 4f_5/2_ are 83.47 eV and 87.1 eV, respectively (Fig. 4c). This suggests that there is no significant doping effect in ML MoS_2_ after plasmonic array fabrication, which is also consistent with the Raman spectra analysis.

**Figure 5:**
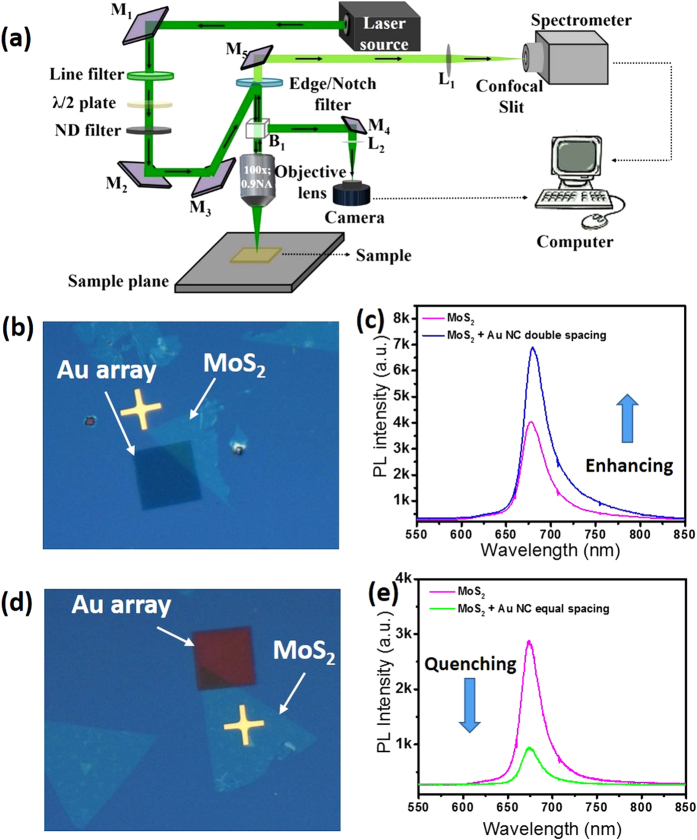
(**a**) Schematic depicting assembled optical set-up for confocal optical microscopy and spectroscopy. (**b**) Optical image of triangular ML MoS_2_ with overlapping region of double spacing Au plasmonic array. (**c**) PL spectra of ML MoS_2_ with and without the Au array shown in (**b**). (**d**) Optical image of triangular ML MoS_2_ with overlapping region of equal spacing Au plasmonic array, and, (**e**) PL spectra of ML MoS_2_ with and without the Au array shown in (**d**).

The excitation wavelength of the laser used for PL measurements was 532 nm (laser power = 50 μW, acquisition time = 1 second, grating = 600) with a 100 ×, 0.9NA objective lens. The excitation laser (from Spectra Physics; maximum output 50 mW, high-quality fundamental transverse electromagnetic mode TEM_00_ beam) is polarized. The polarization state is horizontal with respect to the sample plane (x-y plane). However, even though high magnification (100× lens) optical alignment of polarization axis (i.e. x-axis of sample stage plane at 90° polarization angle) with the axes of the plasmonic lattice array was used, the x-, y- axes of the plasmonic array coupled structure are not aligned perfectly with respect to the polarization axis of the incident excitation due to the manual nature of the alignment procedure. It is assumed that the incident laser polarization (at 90°) axis is along some small, arbitrary angle (ψ°) with respect to the x-axis of the plasmonic Au NC array lattice. To extract the PL modulation of ML MoS_2_ via coupling with the SPR band of Au NCs, different areas of the samples are illuminated using the focused laser beam. The polarization and intensity of the incident laser beam are controlled by engaging a half-wave (λ/2) plate and a neutral density (ND) filter in the illumination path. An appropriate edge filter was also introduced in the collection path for recording the PL spectra. The recorded light can be directed to either a camera or the spectrometer (which are further connected to a computer) to capture the optical image or record the spectra as the case maybe. Other notations are as follows; M_1_, M_2_, M_3_, M_4_, M_5_: mirrors; L_1_ and L_2_: lenses as shown in schematic diagram of the experiment set-up (Fig. 5a)^35^. The optical image of nearly triangular shaped ML MoS_2_ with double spacing array (rectangular shaped, 22 × 22 μm) is shown in Fig. 5b. Optical image of triangular ML MoS_2_ with equal spacing array is shown in Fig. 5d. We have obtained enhancement in PL from double spacing array coupled ML MoS_2_ sample (Fig. 5c and S3, S4), where the stronger ‘A’ exciton emission peak (~679 nm) is observed when compared with pristine ML MoS_2_. Total PL enhancement factor (EF) can be calculated using the formula^36^,^37^: PL EF = (I_patterned_/I_unpatterned_) × (A_o_/A_bare_), where I_patterned_ and I_unpatterned_ are the PL intensities from ML MoS_2_ with and without the Au plasmonic array, respectively. A_o_ represents the excitation area of the laser spot (π × 800^2^ nm^2^) and A_bare_ represents the area of uncovered ML MoS_2_ within the laser spot. The calculated value for PL enhancement factor is approximately 3.5 for the double spacing Au array. We have obtained the completely opposite result from the equal spacing array coupled ML MoS_2_ sample when excited with the same laser power of 50 μW. The PL intensity of ML MoS_2_ from equal spacing array-coupled area has been quenched significantly (4 times) when compared to pristine ML MoS_2_ (Fig. 5e and S5, S6), where the A exciton emission peak is observed at ~680 nm. Thus by manipulating the structural parameters of the plasmonic array, we experimentally demonstrate modulation of the PL intensity from nearly fourfold quenching to approximately threefold enhancement with low laser power excitation (50 μW). Higher laser power excitation might have a larger effect however other effects such as emission from trionic states, hot electron doping from Au NCs and laser induced heating effect^26^,^38^ would also need to be accounted for. These results show that the exciton emission of ML MoS_2_ can be influenced by plasmonic coupling resulting in PL intensity modulation. Please note that the PL intensity for the triangular ML MoS_2_ flakes varies from flake-to-flake at fixed laser power excitation. However, the comparison of PL intensity with and without plasmonic array coupling is done for the same ML MoS_2_ flake at different positions and hence the interpretation of enhancement/quenching of the PL signal should be valid. Please check supporting info (Figures S3–S6) for some more sets of results.

**Figure 6:**
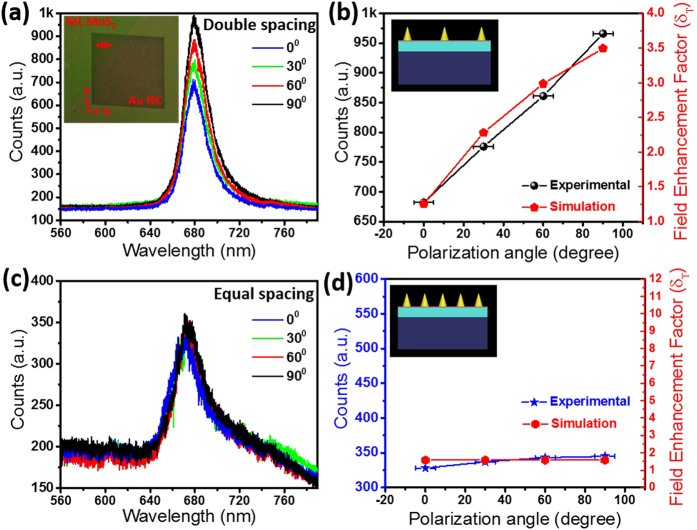
Source polarization angle ( ± ψ°) dependent PL spectra of Au plasmonic array coupled ML MoS_2_/SiO_2_/Si substrates: (**a,b**) PL intensity modulation with different polarization angles for double spacing plasmonic array coupled structure which is shown in inset (**b**). Inset in (**a**) shows an optical image of the double spacing Au NC array coupled with ML MoS_2_ during the PL spectra collection from the red circular point at 90° ( ± ψ°) polarization angle of the laser excitation; (**c,d**) relatively negligible PL intensity variation with polarizing angle for equal spacing plasmonic array coupled structure which is shown in inset (**d**). Calculated total field enhancement factor (δ_T_) for different polarizing angles are co-plotted in (**b,d**).

Enhancement and quenching of PL intensity are further analyzed for varying excitation laser polarization by positioning a λ/2 plate at different angles and the luminescence intensity was monitored using the experimental set-up shown in Fig. 5a. As the double spacing array has asymmetric lateral periodicity, the angle-resolved polarized laser excitation can efficiently tune the induced excitation field in ML MoS_2_ as well as the emission field and the spontaneous emission rate from the ML MoS_2_. Plasmon-induced field in ML MoS_2_ plays an important role during both excitation and emission processes, which we have verified using FDTD simulations. PL spectra from the double spacing and equal spacing plasmonic array coupled ML MoS_2_ at different polarization angles are shown in Fig. 6a and c. The polarization (at 90°) axis of the laser excitation is closely aligned with respect to the x-direction (220 nm spacing) of the double spacing Au NC plasmonic array structure coupled with ML MoS_2_ as shown in the optical image (inset of Fig. 6a). Modulation of PL intensity with polarization angle is observed in double spacing plasmonic coupled ML MoS_2_ (Fig. 6b). On the other hand, PL intensity does not vary significantly for the equal spacing array coupled ML MoS_2_ (Fig. 6d). Due to the manual alignment, we have included error bars in the polarization angle of the experimental data (Fig. 6b,d).

PL intensity of emitters is determined by many factors including the collection efficiency of the spectroscopy setup used for characterization, the excitation rate (γ α |E|^2^; near field intensity enhancement) and quantum efficiency (Q.E.). The intensity of PL (I_PL_) spectra is proportional to γ × Q.E.^25^, which gives I_PL_ = (k × |E|^2^ × Q.E.), where k is a proportionality constant. Induced E- field intensity (|E|^2^) and Q.E. are strongly dependent on the LSPR (Purcell effect). Maximum value of simulated E-field intensity (|E|^2^) of the plasmonic array coupled structure divided with E-field intensity (|E^0^|^2^) without the plasmonic array structure gives the field enhancement factor. Assuming equal contribution of E-field intensity enhancement at excitation wavelength (532 nm) and emission wavelength (680 nm), the total field enhancement factor (δ_T_) can be calculated as follows^27^:


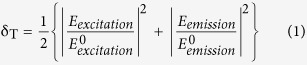


where, E and E^0^ represent the maximum E-fields in the ML MoS_2_ with and without Au NC plasmonic array respectively and subscripts ‘excitation’ and ‘emission’ correspond to the wavelengths of 532 nm and 680 nm respectively. Variation of δ_T_ with polarization angle shows good agreement with modulation of the experimentally obtained PL intensity with polarization angle of the excitation source for the two different array structures (Fig. 6b and d). The effect of different asymmetric ratios is shown in Figures S8 and S9. In the emission band (at 680 nm), which is highlighted in Figure S9a, the double spacing array structure has much higher near E-field intensity value as compared with the rest of the periodic array structures with different asymmetric ratios.

**Figure 7:**
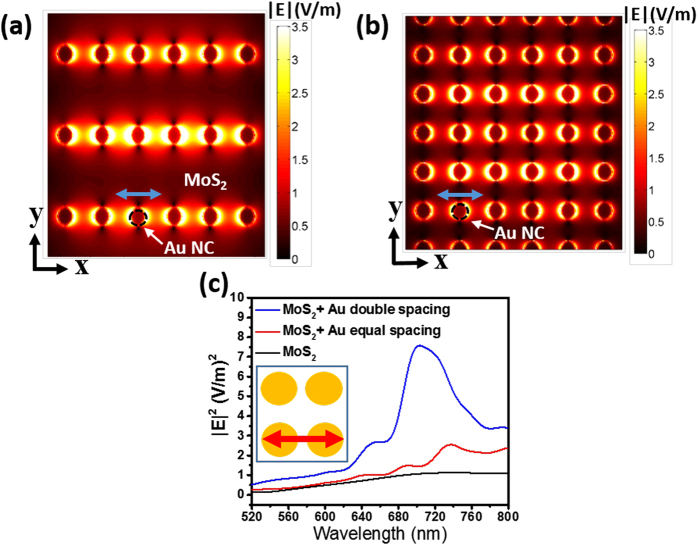
Lumerical FDTD simulation results for Au plasmonic array/ML MoS_2_/SiO_2_(90 nm)/Si structures under plane wave irradiation at 90° incident polarization angle. Near |E| field color plot (at 680 nm) at the interface plane between the plasmonic array and ML MoS_2_: (**a**) for double spacing array, and, (**b**) for equal spacing array. (**c**) Line plot of E-field intensity (|E|^2^) at the center of the spacing between consecutive NCs. Inset shows the E-field direction for double spacing array.

Lumerical finite-difference time-domain (FDTD) simulations were used to calculate E-field intensity distribution for different polarization angles of incident excitation (Figure S7) for the two different array geometries to understand the experimentally observed PL modulation. The details of the simulation set-up are described later in FDTD simulation section. When the polarization axis of the incident excitation is parallel to x-axis of the array (i.e. at 90° polarization angle), the average value of the induced field intensity (|E|^2^) is maximum. The color plot of near E-field distribution (at 680 nm) at the interface plane between the double spacing array and ML MoS_2_ is shown in Fig. 7a. Periodic near E-field distribution (at 680 nm) is observed at the interface plane of the equal spacing array and ML MoS_2_ (Fig. 7b). Line plots of |E|^2^ at 90° polarization angle of excitation for the two different structures along with bare ML MoS_2_ are compared in Fig. 7c. Field enhancement in ML MoS_2_ emission band (~680 nm) is more significant than field enhancement in the excitation band (~532 nm) for the double spacing array coupled structure. Double spacing NC array coupled structure shows significant enhancement of emission field intensity as compared to bare ML MoS_2_ (Fig. 7c), which enhances the PL intensity of ML MoS_2_ as seen in Fig. 5c. Although the equal spacing NC array has slightly higher E-field intensity as compared to ML MoS_2_ in the emission band (Fig. 7c), it suffers from a higher non-radiative rate due to enhanced metal loss (i.e. annihilation of electron-hole pairs and/or excitons due to the Au NCs) which quenches the PL intensity of ML MoS_2_ as seen in Fig. 5e. Slight increment in |E|^2^ value at 543.69 nm corresponding to the Raman wavenumber of 404 cm^−1^ (E^1^_2g_ mode) for the double spacing array coupled structure could be the reason for higher relative intensity ratio for the Raman peaks (E^1^_2g_/A_1g_) when compared to the single spacing array as seen in Fig. 3.

It has been shown that for very low Q.E. 2D materials like ML MoS_2_ the quantum yield enhancement is dependent primarily on the radiative Purcell factor for spontaneous emission^25^,^31^. Low surface coverage, enhancement in the optical field intensity (Fig. 7c) and strong plasmonic coupling in the emission band leading to a significant radiative Purcell factor result in enhanced PL emission for the double spacing array coupled structure. However, the equal spacing plasmonic array coupled structure suffers from a larger shielding effect as compared to the double spacing array due to higher surface coverage, where the emission from ML MoS_2_ will be blocked^32^. Further as mentioned earlier, it is also plausible that the equal spacing array will have a higher non-radiative recombination rate in the emission band due to the loss of^25^ MoS_2_ emission to a higher density of Au NCs (Figure S10). Another factor could be a higher intrinsic non-radiative decay rate since the number of non-radiative decay channels are higher due to the presence of strain in ML MoS_2_ coupled with the equal spacing array, evidence of which is seen in the Raman spectra in Fig. 3d 34. The combined effect of all these factors may be responsible for quenching of the PL emission for the equal spacing array structure in spite of the small enhancement in the optical field intensity with respect to bare MoS_2_ (Fig. 7c). However, more detailed delineation of the reasons behind the PL quenching phenomena needs further studies.

**Figure 8:**
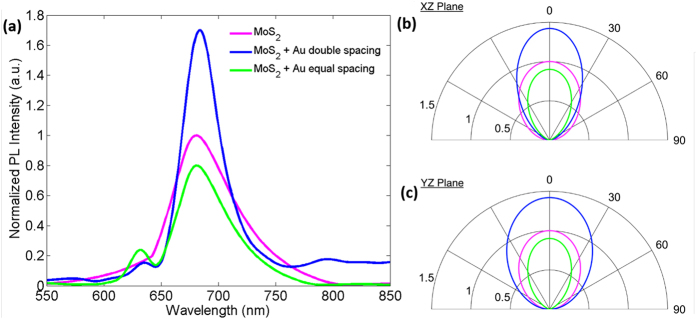
(**a**) Simulation results for the PL spectra of the ML MoS_2_ with and without Au NC arrays. All spectra are normalized with respect to the maximum intensity of ML MoS_2_ to highlight relative enhancement and quenching. (**b,c**) Far-field radiation patterns as a function of angle (degrees) at 685 nm for ML MoS_2_ with Au NC double spacing array (blue), Au NC equal spacing array (green) and without any plasmonic array (magenta).

The ability to control the optical properties of plasmonic nanoresonator coupled TMDCs by applying an electric field in a field effect transistor device^39^ or by using optical pumping^23^,^26^,^27^,^28^ has been demonstrated. In such studies, researchers typically use FDTD method to calculate the field intensities near TMDCs with and without metal nanoparticles and compare them to estimate field enhancement. In this work, we take another path to calculate the PL enhancement. We use Wavenology^40^, which is a commercial 3D full-wave field-circuit co-simulation software package that can solve for circuit and Maxwell’s equations simultaneously on the same grid using FDTD and spectral-element time domain (SETD) methods. Silicon substrate, SiO_2_ and MoS_2_ films are assumed to be 1 μm, 90 nm and 0.7 nm thick, respectively, and cover the region 0 ≤ x, y ≤ 2.5 μm. The dispersive permittivity models for Si and SiO_2_ are taken from ref. 41, whereas complex permittivity model of Au is taken from ref. 42. MoS_2_’s complex electrical permittivity is calculated using our previous report^43^. A broadband wave-port is used to excite the structure from an artificial port, which is placed at x = 0 between the MoS_2_ and Si layers. To guarantee high numerical accuracy, 30 points-per-wavelength sampling density is utilized over the whole simulation domain. We place 400 observers at 2 μm above the MoS_2_ film. Figure 8a shows the average emitted power calculated at these observers as a function of wavelength for 550 nm < λ < 850 nm. Two peaks can be found in the normalized PL intensity at ~630 nm, and ~680 nm, which can be correlated to the B and A excitons of MoS_2_, respectively. Metal nanoparticles indeed influence the PL intensity, i.e., an enhancement is obtained for the double spacing array and quenching is observed for the equal spacing array coupled ML MoS_2_ sample.

To study the effect on the far-field radiation pattern of the two different Au NC periodic array geometries coupled with ML MoS_2_, we assume two dipoles; one along the x-axis and another one along y-axis at the middle region of ML MoS_2_ and calculate the far-field radiation patterns. There are two normalizations considered in the far-field radiation patterns. First, we assume a plane wave incidence and calculate the electromagnetic energy in the middle of the ML MoS_2_ with and without NC array. Then we calculate the far field patterns. These far field patterns are normalized with energy values obtained in the first step. Then the second normalization is done by dividing all the far-field patterns with the pattern at I(θ = 0°). Figure 8b,c show the far-field radiation patterns at 685 nm on the XZ and YZ- plane, respectively. For “ML MoS_2_” and “ML MoS_2_ + Au equal spacing array” cases, the patterns are symmetric and we basically have similar ellipsoidal-like shapes on both planes, where the latter has a smaller maxima, as expected from the results shown in Fig. 8a. “ML MoS_2_ + Au double spacing array” has different patterns on the XZ- and YZ-planes due to changing periodicity along x- and y-directions. On both planes, the maximum intensity is 40% higher than the bare MoS_2_ case.

## Conclusion

In summary, we have demonstrated enhancement (~3.5 fold) and quenching (~4 fold) in PL intensity of ML MoS_2_ by utilizing surface plasmonic resonance (SPR) and manipulating the structural parameters of an Au nanocone plasmonic arrays. Coupling of the SPR mode of the Au arrays provides a mechanism to control the emission of ML MoS_2_. Plasmon induced field in ML MoS_2_ during excitation and emission processes greatly influence the overall emission. We show that PL intensity modulation is controlled via both optical field enhancement and spontaneous emission rate enhancement. Enhancement and quenching of luminescence from plasmonic array coupled ML MoS_2_ structures and its far-field intensity distribution have been studied numerically by optical pumping. This method offers an important way of emission modulation from quenching to enhancement with the opportunity of polarization-based emission control for development of future optoelectronic applications.

## Methods

### Synthesis of ML MoS_2_ on SiO_2_/Si substrate

The CVD growth was performed at atmospheric pressure while flowing ultrahigh purity argon (5 N) gas. Prior to the growth, high purity MoO_3_ (99.5%, Aldrich, 15 mg) powder was placed in a ceramic boat at the center of a 2-inch diameter furnace. In another alumina boat, sulfur (99.95%, Aldrich, 500 mg) powder was placed upstream relative to the gas flow direction at a position 15 cm from the MoO_3_ powder. SiO_2_ (90 nm)/Si substrate was first sonicated in acetone and then in isopropyl alcohol (IPA) for 15 minutes each. Subsequently, it was immersed in a freshly prepared piranha solution (H_2_SO_4_:H_2_O_2_, 3:1) for 1 hour before washing with DI water and finally dried at 90 °C on a hot plate for 15 minutes. The substrate was mounted on top of the boat containing MoO_3_ powder with its polished surface facing downward. It was supported from the bottom by two SiO_2_ coated Si strips^33^. After purging the quartz tube with argon for 15 minutes at 300 standard cubic centimeters per minute (sccm), the reactor was heated to 150 °C for 15 minutes to remove contaminations present in the system. Subsequently, the temperature was ramped to 680 °C at 15 °C minute^−1^ with 10 sccm argon (Ar) flow. Temperature in this step was maintained at 680 °C for 5 minutes. Furnace was then switched off and the reactor was allowed to cool under the same Ar flow rate. When the temperature reached 540 °C, the flow rate of argon was increased to 300 sccm and maintained until the reactor temperature went down to room temperature.

### FDTD Simulation

The simulations were performed using commercial software packages Lumerical FDTD^44^ unless otherwise specified. Normal incidence of polarized plane-wave light was used as a source to irradiate the designed structure to mimic the experimental setup with laser excitation. The reflectance spectra and electric field distribution were recorded simultaneously. Perfect matched layers (PML) were applied at the x- and y- boundaries for 6 × 3 units of double spacing and 6 × 6 units of equal spacing Au NC array coupled structures. PML were also applied at the z boundaries. The thickness of the MoS_2_ was taken to be ~0.7 nm. An additional mesh size of 0.01 nm for the MoS_2_ layer region was utilized making sure that enough mesh points are present inside the material. For the E-field intensity distribution^44^ calculations, we placed the Au array coupled ML MoS_2_ in the xy-plane and the source excitation on top along the z-axis. The span of z-axis was such that the distance between the structure and top z-axis PML boundary was at least half of the maximum wavelength. Wavelength dependent complex permittivity of the materials were used to define their absorption. Lumerical FDTD simulations were performed on a Dell Precision workstation with the following components: Dual Intel Xeon E5 2650V3 processors (10 core) at 2.3 GHz and 128 GB DDR4 RAM.

### Sample fabrication

Large area rectangular plasmonic arrays (22 μm × 22 μm) were patterned with monolayer MoS_2_ on SiO_2_/Si substrate. Raith 150^TWO^ electron beam lithography (EBL) system was used to fabricate the patterns on ML MoS_2_ coated SiO_2_/Si substrate. After monolayer MoS_2_ identification, alignment markers were made by standard electron beam lithography using resists (PMMA and EL-9). The detailed process flow to pattern Au plasmonic array on top of pre-defined markers on ML MoS_2_ is described below:

Resist spinning for plasmonic array: The e-beam dose window was optimized for the following particular combination of bilayer PMMA. Combination of bilayer PMMA. An EBL resist (PMMA 950 K with 2% solid content dissolved in Anisole) was spun at 2500 revolutions per minute (RPM) for 45 seconds and baked 45 seconds and baked on a hot plate at 180 °C for 40 seconds. For coating of the second layer, 4% PMMA 950 K was spun on top of the first layer PMMA at 6000 RPM for 40 seconds followed by a long baking on a hot plate at 180 °C for 90 seconds.

E-beam exposure: Manual write-field alignment technique using pre-defined aligned markers was employed to perform the exposure. The writing field was set at 4000x, 25 μm. Focused beam of electrons, accelerated with an energy of 20 keV and through a 10 μm aperture (beam current: 0.034 nA) was scanned over the sample for writing arrays of equal and double spacing on bilayer PMMA coated samples. Typically, area doses of 320 μC/cm^2^ and 300 μC/cm^2^ were used for equal and double spacing array, respectively.

Development: Dip in an MIBK: IPA mixture (1:3 by volume ratio) for 90 seconds at room temperature and 45–55% humidity conditions followed by rinsing in IPA for 30 seconds and finally blow dry with N_2_ gas.

Metal deposition: Au metal was deposited on top of the patterned substrate using thermal evaporation at a base pressure of 4 × 10^−6^ mbar. Au metal was deposited to obtain a total thickness of ~120 nm at a deposition rate of ~5 nm/minute.

Lift-off: After Au deposition, the sample was immersed in an acetone bath for ~3 hours at room temperature to remove the resist and metal on top of it except the developed plasmonic array area.

## Additional Information

**How to cite this article**: Mukherjee, B. *et al*. Exciton Emission Intensity Modulation of Monolayer MoS_2_ via Au Plasmon Coupling. *Sci. Rep.* **7**, 41175; doi: 10.1038/srep41175 (2017).

**Publisher's note:** Springer Nature remains neutral with regard to jurisdictional claims in published maps and institutional affiliations.

## Supplementary Material

Supplementary Information
