# Supplementary material for: Exciton Emission Intensity Modulation of Monolayer MoS2 via Au Plasmon Coupling
Source: Sci Rep. 2017 Jan 30;7:41175. doi: 10.1038/srep41175 (PMC5278406; doi:10.1038/srep41175)
Supplement: Supplementary Information [file srep41175-s1.pdf]

SUPPLEMENTARY INFORMATION:

Exciton Emission Intensity Modulation of Monolayer MoS_2 via Au Plasmon Coupling

B. Mukherjee[1*], N. Kaushik[1], Ravi P. N. Tripathi[3], A. M. Joseph[1], P. K. Mohapatra[2], S. Dhar[2], B. P. Singh[2], G. V. Pavan Kumar[3], E. Simsek[4] and S. Lodha[1*]

[1] *Department of Electrical Engineering, Indian Institute of Technology Bombay, Mumbai, Maharashtra 400076, India*

[2] *Department of Physics, Indian Institute of Technology Bombay, Mumbai, Maharashtra 400076, India*

[3] *Photonics and Optical Nanoscopy Laboratory, Physics Division and Center for Energy Science, h-cross, Indian Institute of Science Education and Research, Pune 411008, India*

[4] *Department of Electrical and Computer Engineering, School of Engineering and Applied Science, The George Washington University, Washington, D.C. 20052, USA*

*Correspondence and requests for materials should be addressed to B. Mukherjee (Email: bmukherjee@iitb.ac.in) and S. Lodha (Email: slodha@ee.iitb.ac.in)

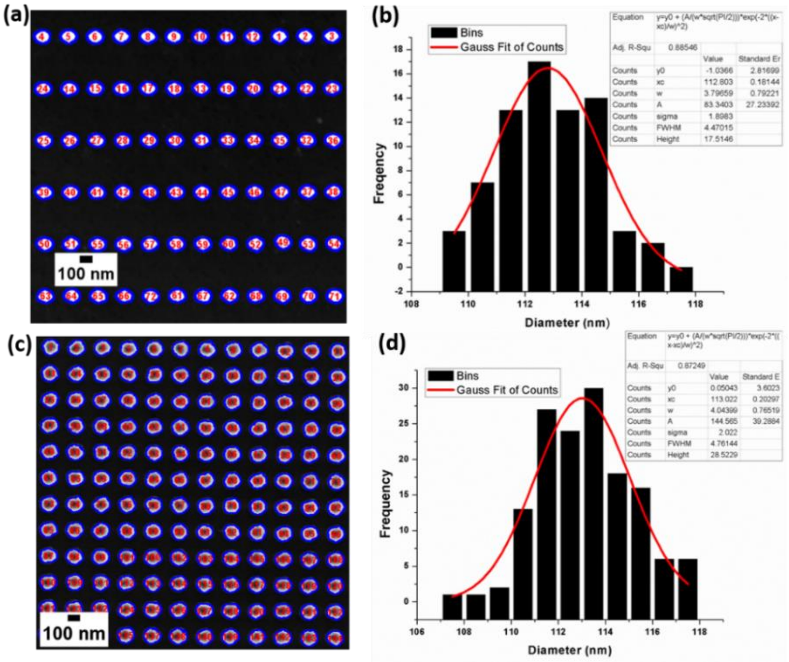

Figure S1. ImageJ analysis of plasmonic array: (a,b) for double spacing array of area 2.4 ×2.4 μm^2; the average bottom diameter of Au NC is 112.8 ± 1.9 nm and (c,d) for equal spacing array of area 2.44 × 2.44 μm^2; the average bottom diameter of Au NC is 113.02 ± 2.02 nm.

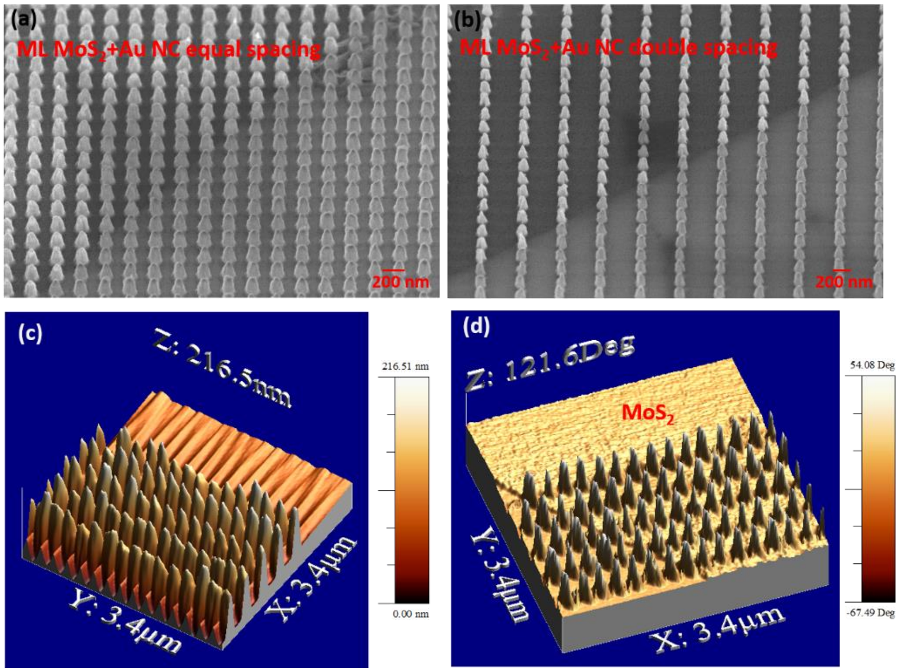

Figure S2. (a,b) A tapping mode 3D Atomic Force Microscopy (AFM) topography image of equal and double spacing Au NC array coupled ML MoS_2 sample, respectively. (c,d) height profile and phase contrast imaging, respectively.

Photoluminescence Enhancement of ML MoS_2 with Au NC array

The two major important factors responsible for PL intensity are $|E|^2$ and quantum efficiency (Q.E.) of the designed structure.

Firstly, consider the near E-field intensity distribution (Figure 7 a,b), which is shown at emission wavelength (680 nm) and the extracted maximum near field intensity value multiplied with Q.E. used to explain PL modulation data (Figure 6 b,d). We don't see significant E-field intensity variation at excitation laser frequency, which we observe at emission frequency (Figure S3). LSPRs of the NC array are mainly responsible for the modification of the localized E-field intensity at emission frequency as compared with only a slight modification at excitation frequency.

Secondly, LSPR offers the ability to control the emission due to high Purcell enhancement by allowing additional local density of states. η and η` represent Q.E.s for bare MoS_2 without and with Au NC plasmonic array, respectively. γ_{rad} and $\gamma_{non\text{-}rad}$ are the radiative and non-radiative decay rates. Q.E. enhancement of such 2D TMDCs decorated with plasmonic array is strongly dependent on the LSPR (Purcell effect), which is explained as follows. [1,2]

$$\eta = \frac{\gamma_{rad}}{\gamma_{rad} + \gamma_{non-rad}}$$

And,

$$\eta` = \frac{\Gamma_{rad} \times \gamma_{rad}}{(\Gamma_{rad} + \Gamma_{non-rad})\times\gamma_{rad} + \gamma_{non-rad}},$$

where Γ_{rad} is the partial Purcell factor leading to radiative decay and $\Gamma_{non\text{-}rad}$ is the remaining component of the Purcell Factor resulting in an effective non-radiative rate due to metal loss. Assuming intrinsic non-radiative rate ($\gamma_{non\text{-}rad}$), which could be defect-mediated non-radiative recombination in ML MoS_2 or strain induced non-radiative decay channel produced during plasmonic array fabrication, is invariant,

$$\text{Q.E. enhancement} = \frac{\eta`}{\eta} = \frac{\Gamma_{rad}}{(\Gamma_{rad} + \Gamma_{non-rad} - 1)\times\eta + 1}$$

The intrinsic Q.E. of monolayer MoS_2 is very low due to defect mediated non-radiative decay channels, typically $\eta = 10^{-3} \sim 10^{-4}$. Thus $\gamma_{non\text{-}rad}$ is orders of magnitude larger than γ_{rad}, which implies firstly, $\gamma_{non\text{-}rad} \gg \Gamma_{rad} \times \gamma_{rad} \gg \gamma_{rad}$ and secondly $\eta \times \Gamma_{non\text{-}rad} << 1$.

Thus the final expression for the quantum yield enhancement becomes

$$\frac{\eta`}{\eta} = \frac{\Gamma_{rad}}{(\Gamma_{non-rad} \times \eta) + 1} = \Gamma_{rad}$$

Hence Q.E. enhancement is linearly proportional to the radiative Purcell factor for the spontaneous emission only. Thus our experimental observation of exciton emission enhancement by Au NC plasmonic array has the important factor to be considered, i.e., the LSPR dependent radiative Purcell factor. Also we have shown that double spacing array has higher SPR coupling with ML MoS_2 than equal spacing array so it further helps to enhance the exciton emission of MoS_2.

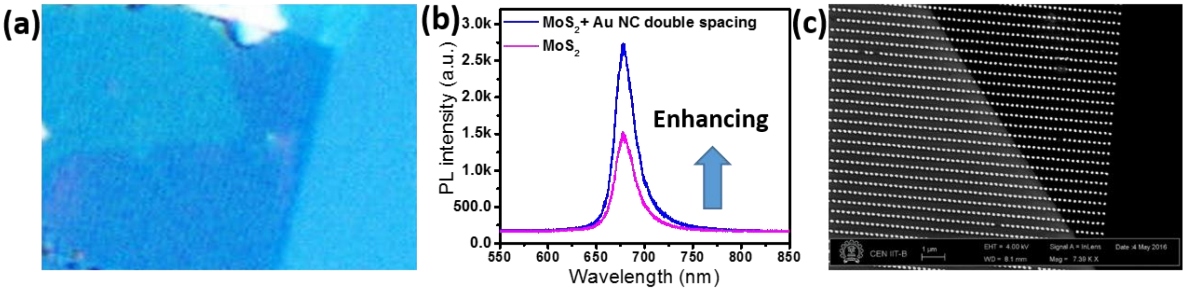

Figure S3. (a,c) Optical and SEM images of monolayer MoS_2 with double spacing Au NC plasmonic array. (b) PL spectra of monolayer MoS_2 with and without double spacing Au NC array.

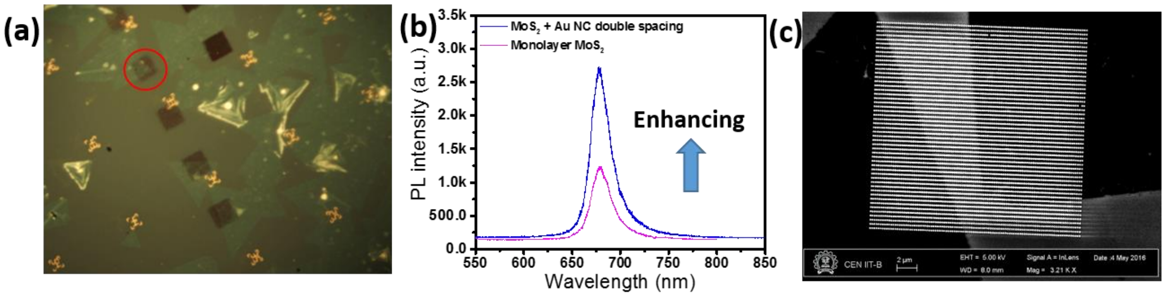

Figure S4. (a,c) Optical and SEM images of monolayer MoS_2 with double spacing Au NC plasmonic array. (b) PL spectra of monolayer MoS_2 with and without double spacing Au NC array. Red circle in (a) indicates the flake where the PL spectra was measured.

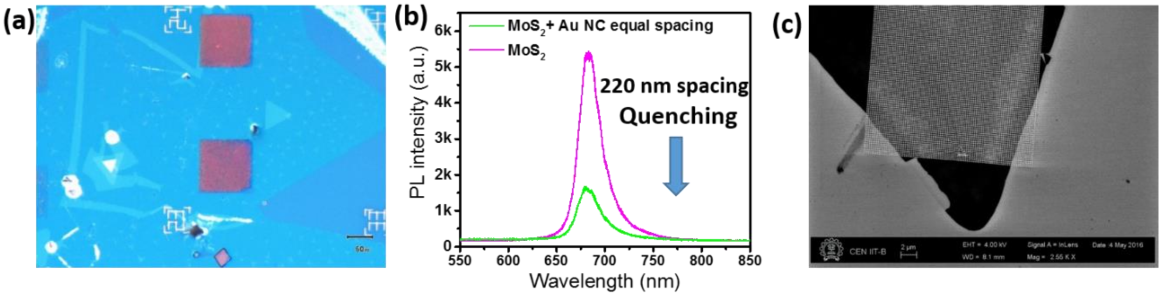

Figure S5. (a,c) Optical and SEM images of monolayer MoS_2 with equal spacing Au NC plasmonic array. (b) PL spectra of monolayer MoS_2 with and without equal spacing Au NC array.

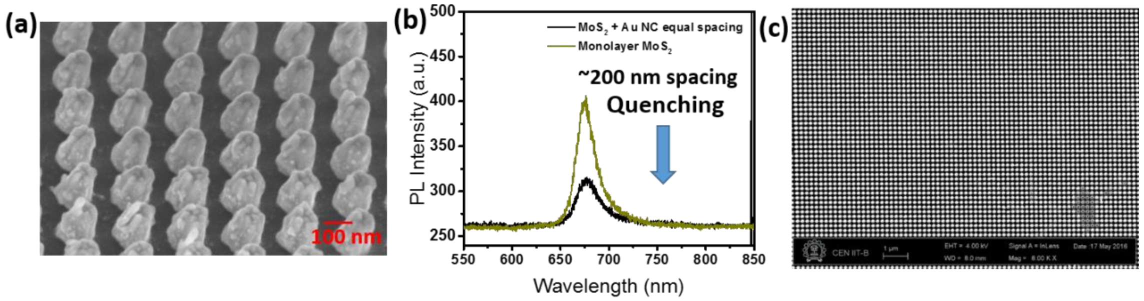

Figure S6. (a,c) SEM images of monolayer MoS_2 with equal spacing Au NC plasmonic array. The periodicity along x- and y- axes is ~200 nm. (b) PL spectra of monolayer MoS_2 with and without equal spacing Au NC array.

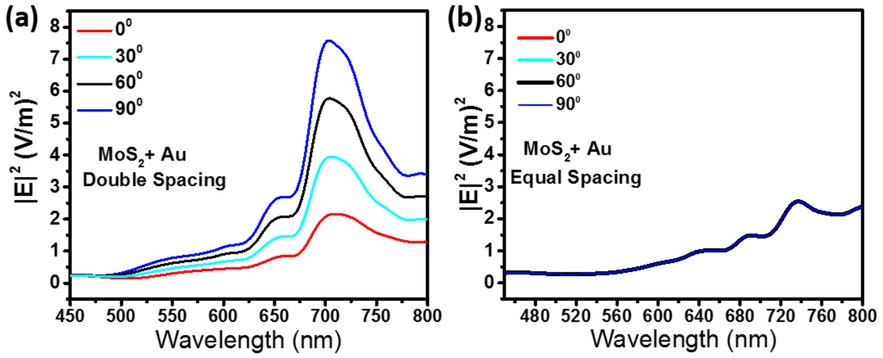

Figure S7. Near E-field intensity plots for different polarization angle of laser excitation: (a and b) line plots of $|E|^2$ for double and equal spacing array coupled ML MoS_2 structure, respectively.

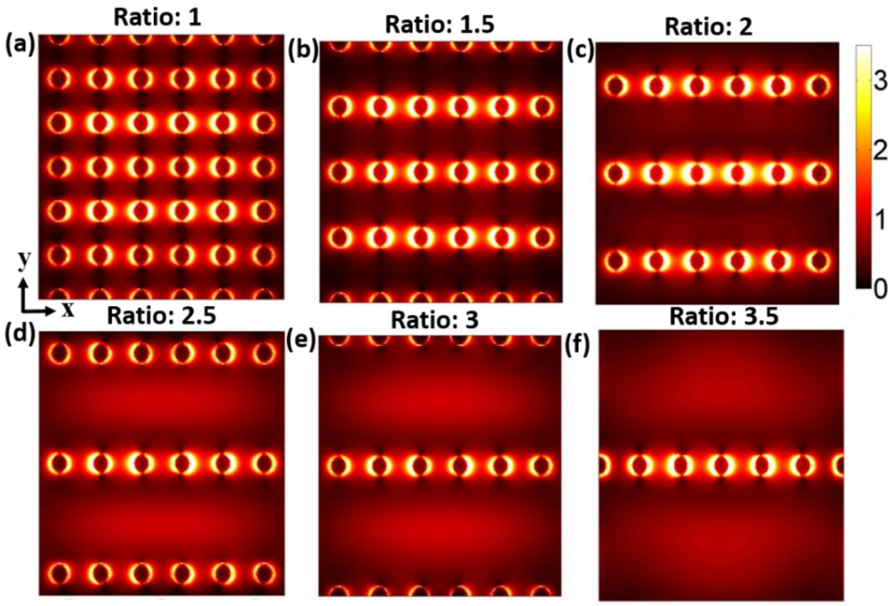

Figure S8. (a-e) Near |E| field color plot at the interface plane between the plasmonic array and ML MoS_2 for different asymmetric ratio (periodicity along y/x axes) array.

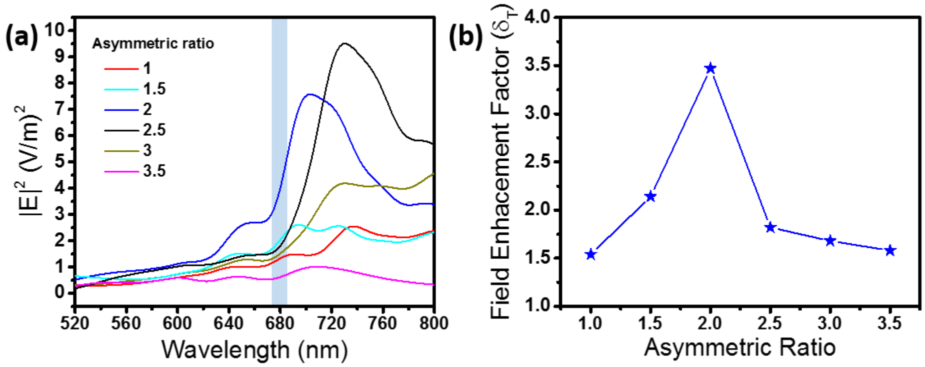

Figure S9. (a) Line plot of near E-field intensity ($|E|^2$) for different asymmetric ratios (periodicity along y/x axes). Polarized plane wave was irradiated along x-axis of the structures. (b) Calculated total field enhancement factor (δ_T) for different asymmetric ratio.

Simulated $\xi = \frac{\gamma'_{rad}}{\gamma'_{rad} + \gamma_{loss}}$ of ML MoS_2 with Au NC arrays:

ξ factor defines the measure of light gain that the dipole source will radiate into the far-field when coupled with the plasmonic NC array and ML MoS_2. For calculating the ξ factor, the dipole source (type: electrical dipole) was placed in the structure such that it had strong interaction with both, the Au NCs and the ML MoS_2. In the ξ factor analysis of the hybrid structure/system

of plasmonic Au NCs-ML MoS_2, the dipole emitter (used as a source in simulation) can decay through an additional path involving radiative and/or non-radiative processes via the local density of states created by the Au NC antennas (metal loss). ξ factor calculates the $\frac{\gamma'_{rad}}{\gamma'_{rad} + \gamma_{loss}}$ value for double and equal spacing NC arrays coupled with ML MoS_2, where γ'_{rad} is the decay rate of excitons to photons that can leave the plasmonic array coupled to ML MoS_2 by radiation and γ_{loss} is the decay rate of excitons that are absorbed or otherwise lost in the system (e.g., photons absorbed by the Au NCs). The dipole source region does not overlap with any active material used in the unit cell of the simulation. During the simulation, power absorbed by Au NC array and ML MoS_2 was monitored and matched with theoretical values. In the analysis of the ξ factor, we have included the contribution from radiative and loss mechanisms while ignoring non-radiative losses due to non-EM processes (e.g. defect and/or disorder mediated losses). The loss term includes the conversion of created excitons into photons that are absorbed by the structure materials. The contribution of non-radiative decay of excitons due to presence of defects or other, similar, non-radiative channels present in the system such as the internal non-radiative recombination of excitons due the low internal Q.E. of ML MoS_2 are neglected in the simulated ξ spectra.

Utilizing a dipole source for the excitation process, ξ spectra were simulated as shown in **Figure S10**. Overall ξ values are slightly higher in the double spacing array geometry as compared with the equal spacing array coupled structure, which could be due to the fact that the equal spacing array coupled structure has higher metal loss as compared with the double spacing array structure.

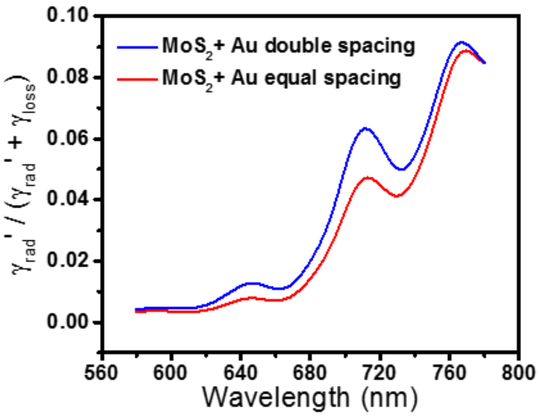

Figure S10. Simulated ξ for double and equal spacing Au NC array coupled with ML MoS_2.

Reference

1. Zhao, W. *et al. Adv. Mat.* **28 (14)**, 2709–2715 (2016). Exciton-plasmon Coupling and Electromagnetically Induced Transparency in Monolayer Semiconductors Hybridized with Ag Nanoparticles.

2. Gao, W. *et al*. Localized and Continuous Tuning of Monolayer MoS_2 Photoluminescence Using a Single Shape-Controlled Ag Nanoantenna. *Adv. Mater.* **28**, 701–706 (2016).
